# Supplementary material for: SensiMix: Sensitivity-Aware 8-bit index & 1-bit value mixed precision quantization for BERT compression
Source: PLoS One. 2022 Apr 18;17(4):e0265621. doi: 10.1371/journal.pone.0265621 (PMC9015158; doi:10.1371/journal.pone.0265621)
Supplement: Attachment — Submitted filename: rebuttal.pdf [file pone.0265621.s001.pdf]

Rebuttal Letter

1 Response to Reviewer 1

We would like to thank Reviewer 1 for the insightful and detailed feedback. Comments and how we address them in our paper are summarized below.

[Comment 1]

- I'm confused by the terms "8-bit index" and "1-bit value". If I am understanding correctly, both 8-bit and 1-bit are quantization strategies on the weights matrices. I'm not sure what the index in "8-bit index" refers to, or why it's different from the value in "1-bit value", other than in bit-width. If the difference between the two strategies is only the amount of quantization (and which layers you apply them to), I would suggest you either name them similarly to reflect this (8-bit value and 1-bit value quantization), or just refer to them as 8-bit quantization and 1-bit quantization.

[Response to comment 1]

Q. About the difference between the index quantization and the value quantization.

R. The main difference between the 8-bit index quantization and the 1-bit value quantization depends on whether there is a de-quantization process, in addition to the bit sizes. The 1-bit value quantization does not have a de-quantization process, and all weights are quantized to ± 1 and propagated to the next layer directly. However, the 8-bit index quantization has a de-quantization process where FP32 values are first quantized to INT8 indices (we call them indices because they are not the final values that are propagated to the next layer) and then de-quantized back to FP32 values before propagated to the next layer. In the revised paper, we added a precise description of the difference between the value and the index quantization to lines 343-345.

[Comment 2]

- In table 1, you state that matrix Wldq is an "8-bit index-de-quantized weight matrix of the layer l". However, my understanding is that your de-quantization process takes a matrix of INT8s and (with the min and max) regenerates an approximation of the original matrix of 32FPs. Shouldn't this matrix contain 32-bit values? If not, why not? This also leads to confusion in 8-bit de-quantization, where the de-quantized values are weight e.g. -1.28, 0.005, 1.00, and 1.27, but Wldq is said to be an 8-bit matrix. How can you store a value of -1.28 inside a INT8? Line 312 then describes it as an 8-bit clip function, but if the input to alg. 4 was indeed an 8-bit signed integer, the clip function should have no effect. The only way I can make sense of this is if x is actually a FP32, but that doesn't agree with what you've previously written about Wldq.

[Response to comment 2]

Q. About the definition of the W_{dq}^{l} matrix.

R. It is right that W_{dq}^{l} is the de-quantized FP32 weight matrix which contains FP32 values, and we stated this matrix as "8-bit index-de-quantized weight matrix of the layer l", which might bring misunderstandings to readers. In the revised paper, we revised the definitions of W_{q}^{l} and W_{dq}^{l} in Table 1 to make the statement more clear.

[Comment 3]

- Line 268: "8-bit index quantization consists of two steps: quantization and de-quantization." This sentence is very confusing. I understand that both quantization and de-quantization are elaborated on later, but I find it confusing to have step 1 of quantization be its self, and step 2 be undoing its self. I think what you're getting at is something like, "In order to utilize 8-bit quantization, SensiMix is able to quantize 32FP values to 8-bit int values, and de-quantize the values back to 32-bit.", but elaboration would make this clearer.

[Response to comment 3]

Q. About the elaboration of the process of the 8-bit index quantization.

R. It is right that the quantization step quantizes FP32 values to INT8 values (indices), and the de-quantization step de-quantizes INT8 values back to FP32 values. In the revised paper, we added a precise elaboration about this part to lines 279-281.

[Comment 4]

- I have some issues with the differentiability of your clipping functions. In the 8-bit clip, if the input are FP32 values, values outside of the range (-128,127) will be clipped to -128 and 127. However, the clip function is undifferentiable at this values. Why is this not a problem? You mention in the 1-bit clip section that most values are near zero, and are unlikely to be near the clipping limits, but I don't have a similar kind of intuition about the 8-bit clipping. I would appreciate if you would elaborate about why both clip functions being undifferentiable at their min and max is not a problem (or if it is, explain).

[Response to comment 4]

Q1. About the intuition of the 8-bit clip function.

R1. We use the 8-bit clip function to replace the 8-bit round function in the training process to solve the zero gradient problem of the 8-bit round function. The derivative of the 8-bit round function is equal to zero in almost all ranges which disables the training of the model using gradient descent-based training methods, while replacing it by the 8-bit clip function enables the model training.

Q2. About the problem that values outside of the range (-128,127).

R2. In the 8-bit index quantization, FP32 weights in a weight matrix are divided into 256 ranges which are temporarily stored in INT8 numbers (indices), and then we use these INT8 numbers, the minimum weight, and the maximum weight to approximate the original FP32 weight matrix (de-quantization step). Note that in Equation 1, all original FP32 weight values are mapped into range [-128, 127], which means all FP32 weights are updated. In the revised paper, we added statements about this in lines 335-337 to make it more clear.

[Comment 5]

- Figure 3. I'm really not sure what this figure is trying to convey. Shouldn't the weights after ABWR be clustered at -1 and 1? To my understanding of ABWR, the left and right subfigures seem swapped; the values before ABWR should be clustered around 0, and afterwards they should be clustered around -1 and 1. Could you explain why Fig 3 shows what it does?

[Response to comment 5]

R. The order of the figures in Figure 3 was wrong, we corrected the order of the figures in the revised paper.

[Comment 6]

- 392: Figure 4 seems to show a layer FP32 FFN being converted into a 1-bit FFN after 1 epoch, but that doesn't really agree with your description of adding MP layers to the bottom (I assume the bottom of the existing MP encoder layers, but you don't specify this). Which is correct for ILF?

[Response to comment 6]

R. It is right that the "bottom" represents the bottom of the existing MP encoder layers. To make the statement more clear, we revised the lines 407, 408, 410, and 411 which give a more precise description of ILF in the revised paper.

[Comment 7]

- Line 43: you might want to describe 1-bit quantization here, or cite a description. I assume it quantizes values to -1 or 1, but a reader could easily assume it quantizes it to 0 or 1. "For the 1-bit quantization, we apply standard 1-bit quantization." This sentence adds nothing, either explain what 1-bit quantization is, or remove this sentence.

[Response to comment 7]

R. We removed the sentence in the revised paper.

[Comment 8]

- Line 146: suggest changing "low-bit numbers" to something like "reduced precision numbers", or "smaller bit-width integers", since low-bit is ambiguous, and to my ears "low-bit" refers to the position of a bit, not the width of an integer.

[Response to comment 8]

R. We changed the words "low-bit numbers" to "smaller bit-width integers" in the revised paper.

[Comment 9]

- 452: a table link is broken. 520: broken table link.

[Response to comment 9]

R. We fixed the broken reference links in the revised paper.

2 Response to Reviewer 2

We would like to thank Reviewer 2 for the high-quality review and constructive comments. Comments and how we address them in our paper are summarized below.

[Comment 1]

- 1) Outside the BERT domain there seems to be a body of literature on Mixed Precision Quantization based on the sensitivity of the outputs. e.g. these came up with a simple search: [5, 4]
  2) There also seems to be more literature on quantization of BER T models e.g. [3, 2, 1, 6].
  Could the authors elaborate more on this to stress their novelty?

[Response to comment 1]

Q1 About the contribution and novelty of our paper.

R1 Our main claim is to compress the BERT model to a small one while maintaining its accuracy, and we have the following contributions:

- We discover the sensitivity of different modules of BERT. We demonstrate that the encoders close to the input layer are more sensitive than those near to the output layer in BERT, and Self-Attention layer is more sensitive than feed-forward network (FFN) in an encoder.
- We effectively compress the model considering the sensitivity of different modules. We compress the sensitive parts of BERT using 8-bit index quantization and insensitive parts using 1-bit value quantization, which maximizes the compression rate while minimizing the accuracy degradation caused by quantizing sensitive modules to low bits.

- We propose three 1-bit quantization-aware training methods: Absolute Binary Weight Regularization (ABWR), Prioritized Training (PT), and Inverse Layer-wise Fine-tuning (ILF). They overcome the challenges of training 1-bit quantization method, improving the accuracy of the model.
- We introduce a fast inference strategy of SENSIMIX on an NVIDIA GPU. We apply FP16 general matrix multiplication (GEMM) and XNOR-Count GEMM to replace the original GEMM for the 8-bit and the 1-bit quantization parts of the model, respectively, to achieve fast inference speed.

Q2 About comparisons with other mixed precision quantization methods.

R2 We compared our method to Q8BERT which is an effective quantization method for BERT. Other methods are excluded for the following reasons. First, there are difficulties when comparing our method to quantization methods designed on special hardware because the performance changes when the hardware environment changes. Second, methods that are designed for other models (MLP, CNN, and RNN) are not directly applicable to BERT, such as [5] and [4]. However, we agree that comparing to other mixed precision BERT quantization methods is one of the next steps. We added the comment in line 635 in the revised paper.

[Comment 2]

- Line 452 and 520, 538 reference to Table is missing.

[Response comment 2]

R. We fixed the broken reference links in the revised paper.

[Comment 3]

- In the questions outlined in Experiments, the section numbering is missing.

[Response to comment 3]

R. In the revised paper, we removed the section numbers in the experimental questions and added the corresponding question numbers to the title of each section.

3 Response to Reviewer 3

We would like to thank Reviewer 3 for the insightful and detailed comments. Comments and our responses are summarized below.

[Comment 1]

- The term BERT should be expounded first before first time usage.

[Response to comment 1]

R. In the revised paper, we added an introduction of BERT in the first subsection of Related Works (lines 84-92) to expound the term BERT.

[Comment 2]

- The introduction was well prepared (well done!)

[Response to comment 2]

R. Thank you for your kind comment.

[Comment 3]

- The section on related works should be expanded.

[Response to comment 3]

R. We added an introduction of BERT to the first subsection of Related Works (lines 84-92). This subsection expands the related works and provides a clear elaboration of BERT to readers.

[Comment 4]

- The section "Related Work" should be "Related Works".

[Response to comment 4]

R. We revised the term "Related Work" to "Related Works" in the revised paper.

[Comment 5]

- The proposed method SENSIMIX should be expanded to include algorithmic description in form of flowhart or pseudocode. This is necessary to see the actual operation of the SENSIMIX

[Response to comment 5]

R. In the revised paper, we added a pseudocode algorithm of SENSIMIX (Algorithm 1) in Proposed Method.

[Comment 6]

- The authors did nice work with textual description, but the work should be expanded to include diagramatic illustrations

[Response to comment 6]

R. In the revised paper, we added a pseudocode algorithm of SENSIMIX (Algorithm 1) and a figure (Fig 3) on the process of the 8-bit index quantization in Proposed Method to expand diagramatic illustrations of our method.

[Comment 7]

- Is there anyway that Genetic Algorithm (GA) can be used for model reduction in this work ?

[Response to comment 7]

R. We have not combined Genetic Algorithms with our method. However, we added it to our future work to lines 636-637 in the revised paper.

[Comment 8]

- Both 8-bit and MP encoders should be described with a flowchart or pseudocode.

[Response to comment 8]

R. In the revised paper, we added a pseudocode algorithm of SENSIMIX (Algorithm 1) to Proposed Method which includes the algorithms of 8-bit and MP encoders. Furthermore, we added a figure (Fig 3) on the process of the 8-bit index quantization to Proposed Method to give a diagramatic explanation of the 8-bit encoder.

[Comment 9]

- How is FB32 matrix in line 276 represented ? (In terms of matrix)

[Response to comment 9]

R. The FP32 matrix in your comment is the full-precision weight matrix of each layer in the model (corresponding to W^l_{fp} in Table 1). In the revised paper, we gave a more clear elaboration to lines 289-290.

[Comment 10]

- Why the use of the method in Equation 6? Is it better than ADAM method, or SGM?

[Response to comment 10]

R. Equation 6 is a basic training rule to solve the zero gradient problem of the binary function; other gradient descent-based update rules (e.g., Adam) can be used as well. To make the statement more clear, we added an explanation of Equation 6 to lines 367-369 in the revised paper.

[Comment 11]

- In overall, this is a very rich article and well written and described. The authors should include more diagrams and include another section on the description of the computational platform (software) used.

[Response to comment 11]

R. Thank you for your valuable comments. In the revised paper, we added a pseudocode algorithm of SensiMix (Algorithm 1) and an illustration figure of the process of the 8-bit index quantization (Fig 3) in Proposed Method, and we added a description of our software environment to lines 530-531.

References

[1] Fan, C.: Quantized transformer

[2] Jin, J., Liang, C., Wu, T., Zou, L., Gan, Z.: KDLSQ-BERT: A quantized bert combining knowledge distillation with learned step size quantization. CoRR **abs/2101.05938** (2021). URL https://arxiv.org/abs/2101.05938

[3] Kim, S., Gholami, A., Yao, Z., Mahoney, M.W., Keutzer, K.: I-bert: Integer-only bert quantization (2021)

[4] Lee, D., Cho, M., Lee, S., Song, J., Choi, C.: Data-free mixed-precision quantization using novel sensitivity metric. CoRR **abs/2103.10051** (2021). URL https://arxiv.org/abs/2103.10051

[5] Yuan, Y., Chen, C., Hu, X., Peng, S.: Evoq: Mixed precision quantization of dnns via sensitivity guided evolutionary search. In: 2020 International Joint Conference on Neural Networks (IJCNN), pp. 1–8 (2020). DOI 10.1109/IJCNN48605.2020.9207413

[6] Zhao, Z., Liu, Y., Chen, L., Liu, Q., Ma, R., Yu, K.: An investigation on different underlying quantization schemes for pre-trained language models (2020)
