# Supplementary material for: SensiMix: Sensitivity-Aware 8-bit index & 1-bit value mixed precision quantization for BERT compression
Source: PLoS One. 2022 Apr 18;17(4):e0265621. doi: 10.1371/journal.pone.0265621 (PMC9015158; doi:10.1371/journal.pone.0265621)
Supplement: Attachment — Submitted filename: rebuttal.pdf [file pone.0265621.s002.pdf]

Rebuttal Letter

1 Response to Reviewer 2

We would like to thank Reviewer 2 for the insightful and detailed feedback. Comments and how we addressed them in our paper are summarized below.
[Comment]

- Unfortunately the authors have not addressed my concerns with regards to comparison with other quantization methods, making it very difficult to judge the actual novelty of the paper. If an experimental evaluation is not possible, I think there should be at least a full literature review in the background section in quantization methods for neural nets. Such a literature review should explain the differences with each one, why it wasn't used for comparison, and why it SensiMix is novel.

[Response to comment]

- We included a comprehensive literature review in our Related Works section based on the papers you suggested (lines 183-226). We summarized each paper included and explained SensiMix's strengths compared to these works. We believe this revision would help clarify the novelties of SensiMix.

2 Response to Reviewer 4

We would like to thank Reviewer 4 for the high-quality review and constructive comments. Comments and how we address them in our paper are summarized below.
[Comment]

- The authors propose an acceleration of the BERT family of models using quantization techniques. Although the contribution is not difficult to pursue, the results are promising in practice. Therefore, I believe that the paper has merit to be accepted in a multidisciplinary journal such as Plos One.
  I suggest introducing and discussing recent references on how to increase the computational efficiency of BERT from different perspectives. Some examples are:
  - BERT Pre-training Acceleration Algorithm Based on MASK Mechanism
  - Hardware Acceleration of Fully Quantized BERT for Efficient Natural Language Processing
  - Plug-Tagger: A Pluggable Sequence Labeling Framework Using Language Models
  - A Comprehensive Survey on Training Acceleration for Large Machine Learning Models in IoTs

[Response to comment]

- Reflecting your suggestion, we included a brief introduction of effective compression techniques on BERT from different perspectives in the Introduction section (lines 24-30).
